# First person – Jing Liu

**DOI:** 10.1242/bio.053249

**Published:** 2020-06-01

## Abstract

First Person is a series of interviews with the first authors of a selection of papers published in Biology Open, helping early-career researchers promote themselves alongside their papers. Jing Liu is first author on ‘Advanced oxidation protein products change biological behaviors of rat endometrial epithelial cells by activating ERK/P38 signaling pathways’, published in BiO. Jing is a doctor in the lab of Yali Song at the Center for Reproductive Medicine, Department of Obstetrics and Gynecology, Nanfang Hospital, Southern Medical University, Guangzhou, China, investigating whether AOPPs could induce rEECs proliferation and migration, and inhibit apoptosis by activating ERK/P38 signalling pathways.


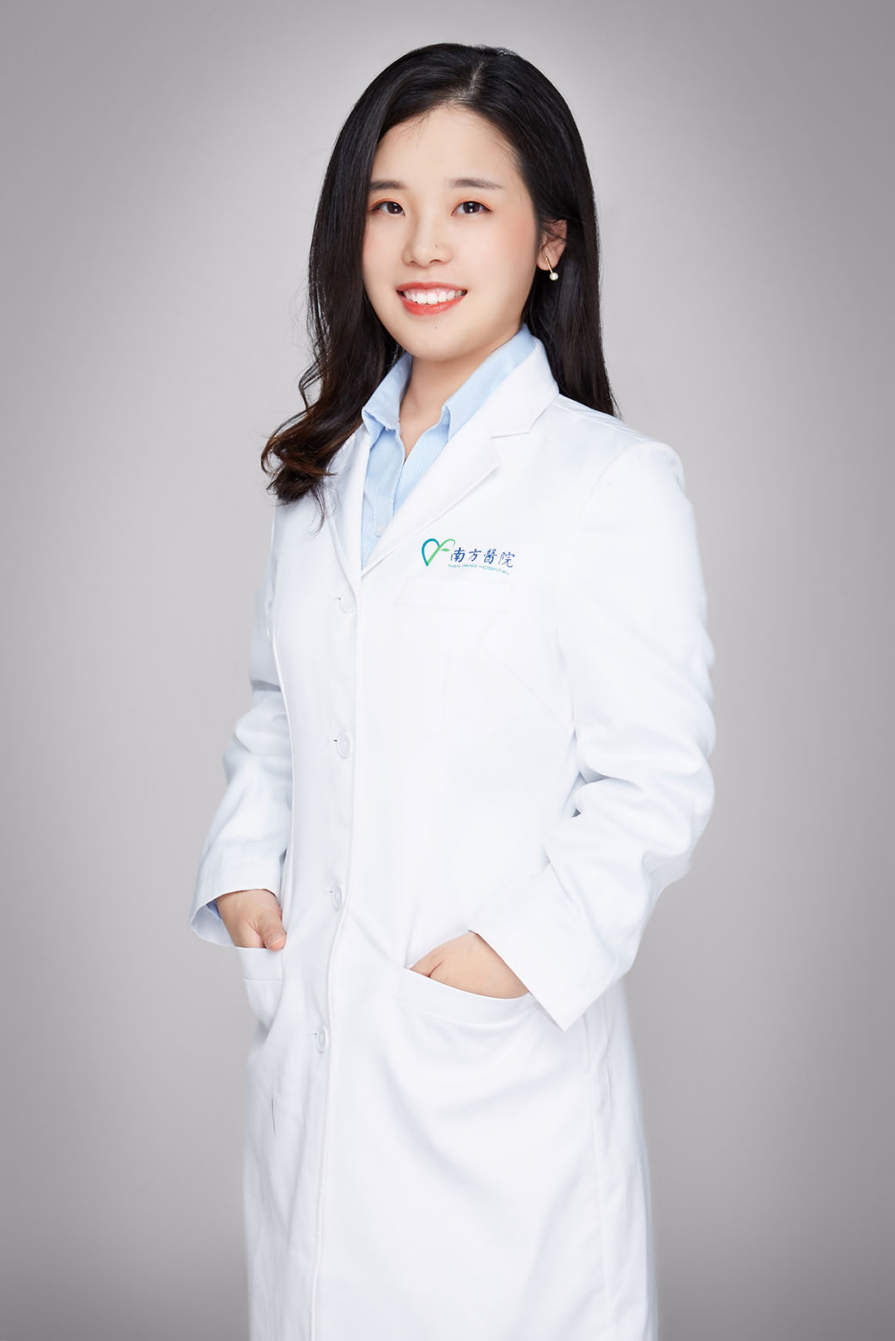


**Jing Liu**

**What is your scientific background and the general focus of your lab?**

While studying for my doctorate, I explored the correlation between oxidative stress and endometriosis under the guidance of Dr Quan and Dr Song. I tried to investigate if advanced oxidation protein products (AOPPs), as the products and triggers of oxidative stress, could affect the biological behaviours of endometrial cells and further promote the development of endometriosis pathogenesis. Over the years, Dr Quan has focused on the pathogenesis of female infertility and the mechanism of repeated implantation failure in *in vitro* fertilization. Dr Song pays close attention to the effects of oxidative stress on female reproductive health.

**How would you explain the main findings of your paper to non-scientific family and friends?**

In the body, oxidation and anti-oxidation are kept in balance. When the balance is broken down, the organism will fail ill. AOPPs are the products of oxidative stress and can trigger oxidative stress. Our research found that overabundant AOPPs could change the biological behaviour of rat endometrial epithelial cells (rEECs) proliferation, migration and apoptosis. At the same time, the production of oxidative stress in rEECs ROS and nitrites both increased, and signalling pathways also were activated. We also confirmed in animal models that AOPPs could promote ectopic endometrial tissue growth by inducing endometrial cell proliferation and migration.

**What are the potential implications of these results for your field of research?**

Abnormal cell behaviours are closely related with the occurrence of various diseases. Increased proliferation and migration of endometrial epithelial cells could contribute to the progression of endometriosis. Previous studies have stated that the accumulation of AOPPs in body fluid were associated with endometriosis. In our research, AOPPs could induce rEECs proliferation and migration by activating ERK/P38 signalling pathways, and promote the growth of ectopic endometrial tissue. These findings showed that the accumulation of AOPPs could contribute the development of endometriosis pathogenesis by activating ERK/P38 signalling pathways.

**What changes do you think could improve the professional lives of early-career scientists?**

When we focus on our professional research, we should keep a sharp eye on the newest research progress and research techniques in various fields. New research techniques can provide us with creative experiment design. To open our eyes to different fields would help us with active thinking. In the future, multidisciplinary research will be the mainstream and the birthplace of great breakthroughs.

**Figure:**
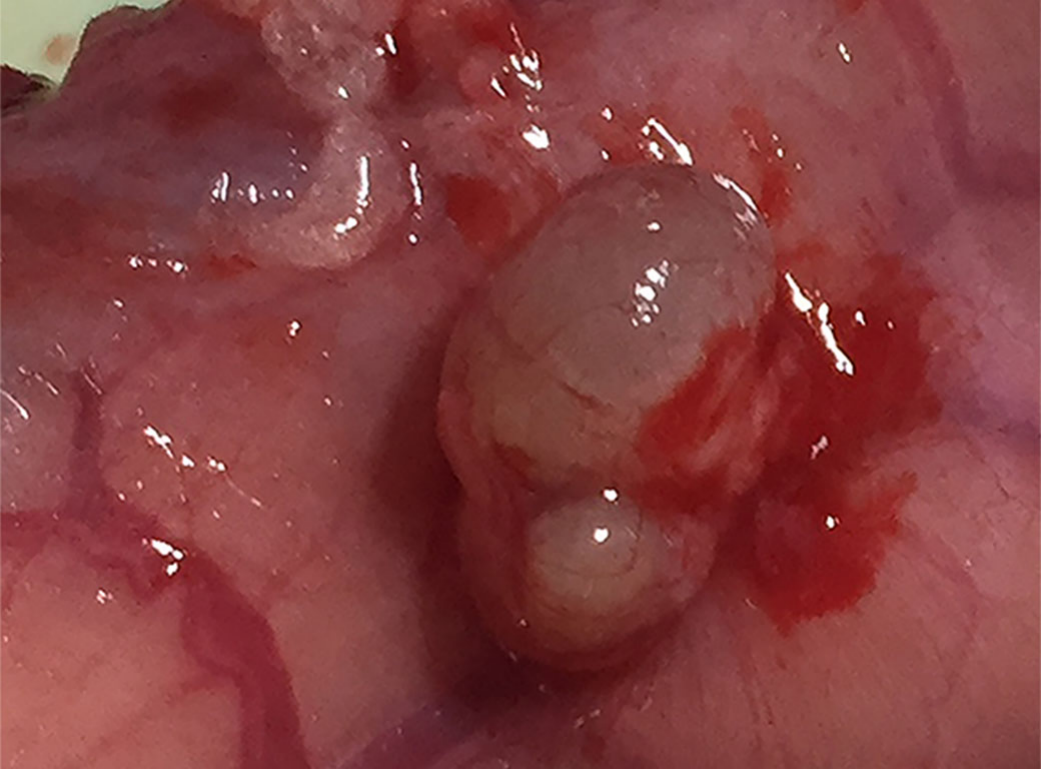
**AOPPs promoted the development of ectopic endometrium tissue.**

“In the future, multidisciplinary research will be the mainstream and the birthplace of great breakthroughs.”

**What's next for you?**

I plan to study the correlation between AOPPs and human endometrial cells further, and try my best to explore the effects of AOPPs on the endometriosis-related infertility. As a clinical doctor, I also need improve my clinical skills in assisted reproductive technology and combine basic research with clinical problems to help more women that are infertile.
